# Prevalence of perinatal asphyxia in East and Central Africa: systematic review and meta-analysis

**DOI:** 10.1016/j.heliyon.2020.e03793

**Published:** 2020-04-26

**Authors:** Yinager Workineh, Ayele Semachew, Emiru Ayalew, Worku Animaw, Mulat Tirfie, Minychil Birhanu

**Affiliations:** a Department of Child Health Nursing, College of Medicine and Health Sciences, Bahir Dar University, Ethiopia; b Department of Adult Health Nursing, College of Medicine and Health Sciences, Bahir Dar University, Ethiopia; c Department of Nutrition, College of Medicine and Health Sciences, Bahir Dar University, Ethiopia

**Keywords:** Public health, Critical care, Pediatrics, Intensive care medicine, Clinical research, Asphyxia, East and Central Africa, Perinatal, Pooled prevalence

## Abstract

**Background:**

Birth asphyxia leads to about 4 million neonatal deaths every year around the globe. But, the pooled prevalence of asphyxia was not yet collated in East and Central African countries. Hence, this systematic review and meta-analysis aimed to determine the pooled prevalence of perinatal asphyxia in Central and East Africa.

**Methods:**

PubMed, Google Scholar, Science Direct, Africa Index Medicus, Africa Journal Online, Excerpta Medica Database, and Cochrane Library databases were searched. All necessary data were extracted using a standardized data extraction format. Data were analyzed using STATA 14 statistical software. A heterogeneity of studies was assessed using the I^2^ statistics. Publication bias was checked by using a funnel plot and Egger's regression test. A random-effect model was computed to estimate the pooled prevalence of perinatal asphyxia.

**Results:**

Thirteen full-text studies were included in the present meta-analysis. The pooled prevalence of perinatal asphyxia in this study was 15.9% (95%CI: 10.8, 21.0% [I^2^ = 94.6, p = 0.000]). Regional subgroup analysis indicated that the pooled prevalence of perinatal asphyxia was 18.0 % (95%CI:11.4, 26.7% [I^2^ = 96.00, p = 0.000]) and 9.1 % (95%CI:2.0, 16.2% [I^2^ = 90.80, P = 0.000]) in East and Central African countries respectively. Similarly, the level of perinatal asphyxia was varied based on asphyxia measuring tools. But the trim fill analysis pointed that there was no difference in the pooled prevalence of perinatal asphyxia in this study.

**Conclusion:**

The pooled prevalence of perinatal asphyxia was high in the current study. It had also substantial variation across the regions and measuring tools. Therefore, there is a call to reduce the high burden of this problem in the region.

## 1 Introduction

Perinatal asphyxia is defined as a condition that leads to progressive hypoxemia, hypercapnia, and metabolic acidosis with multi-organ failure [1]. Perinatal asphyxia is also defined as the inability of a newborn to initiate and sustain adequate respiration after delivery [2]. According to the American College of Obstetricians and Gynecologists, and the American Academy of Pediatrics, a neonate is labelled to be asphyxiated if (a) umbilical cord arterial pH < 7; (b) Apgar score of 0–3 for longer than 5 min; (c) neonatal neurological manifestations (seizures, coma or hypotonia); and (d) multisystem organ dysfunction (cardiovascular, gastrointestinal, hematological, pulmonary or renal system) [3].

Globally, 2 to 10 per 1000 term newborns faced perinatal asphyxia [4]. The report of the World health organization (WHO) indicated that 4 million neonatal deaths occur yearly due to birth asphyxia [5]. The incidence of birth asphyxia in most developed countries accounts less than 0.1% of newborn deaths. But, in developing countries, it ranged from 4.6/1000 to 7–26/1000 live births [6].

More than 25.0% of the world's newborn deaths have occurred in Africa. Of those, birth asphyxia accounts 24.0%. From 20 countries in the world with the highest risk of neonatal death, 75.0 % are in Africa [7]. Birth asphyxia, infections and complications of preterm birth together account 88.0% of newborn deaths in Africa. In Sub-Saharan Africa, birth asphyxia brought 280,000 deaths of the newborn in the first day of life [8]. The incidence of asphyxia in East, Central, and Southern Africa was 22.0% [9].

The common causes of birth asphyxia are umbilical cord problems, ruptured uterus, preeclampsia/eclampsia, placental abruption, placenta previa, anesthesia mistakes, oligohydramnios, premature rupture of the membranes, premature birth, prolonged and arrested labor, uterine hyper-stimulation, fetal stroke, post-maturity syndrome and delayed emergency cesarean section [10, 11].

Hypoxia, hypercarbia, acidosis, hypotension, and ischemia are the immediate result of birth asphyxia. In the long run, it also brings cerebral palsy, seizure disorders, motor disorders, developmental delays, speech delays, learning disabilities, behavioral and emotional disorders, hearing impairments, visual and feeding impairments [12, 13, 14].

The burden of perinatal asphyxia is critical in Sub-Saharan Africa in general, in East and Central Africa in particular. This problem is high in this region due to poor obstetrics coverage, equity and quality because of gaps in local health financing models, inaccessible health facilities, socio-cultural norms, low literacy levels, shortage in health workers and supplies and poor health care spending [15, 16, 17]. Specially, reproductive health services utilization (facility deliveries, skilled delivery assistance and 4^+^ antenatal visits) was lower in East and Central Africa as compared to the other region of Sub-Saharan Africa countries [18].

Despite the existence of the agreement of the international community to reduce neonatal mortality to at least 12 deaths per 1,000 live births by 2030 [19], the pooled prevalence of perinatal asphyxia was not investigated by researchers in East and Central African countries. Previous studies on this issue were fragmented and inconsistent. Even, the prevalence of perinatal asphyxia varied from 3.1% [20] to 32.8% [21] across this geographical setting and time periods. Therefore, this study aimed to determine the pooled prevalence of perinatal asphyxia in East and Central African counties by using systematic review and meta-analysis.

## 2 Methods

### 2.1 Protocol and registration

The results of this review were reported based on the Preferred Reporting Items for Systematic Review and Meta-Analysis statement (PRISMA) guideline [22]. It is not registered in the Prospero database.

### 2.2 Eligibility criteria

The inclusion criteria were: 1) hospital-based studies, 2) observational (cross-sectional, case control and cohort) studies reported the prevalence of perinatal asphyxia among newborns, 3) studies conducted in East and Central African countries, 4) studies published in English, 5) studies used the Apgar score for asphyxia diagnosis, and 6) studies available at the electronic source before September 2019.

On the other hand, citations without abstract and/or full-text, anonymous reports, editorials, and qualitative studies were excluded from the analysis.

### 2.3 Information sources

PubMed, Google Scholar, Science Direct, African Index Medicus, Africa Journal Online, EMBASE and Cochrane Library were accessed. Articles with incomplete reported data were handled through contacting corresponding authors.

### 2.4 Searching strategy

The core search terms and phrases were “perinatal”, “birth”, “parturition”, “newborn”, neonate”, “asphyxia”, suffocation”, “respiratory distress syndrome”, “Tanzania”, “Burundi”, “Rwanda”, “Uganda”, “Sudan”, “Ethiopia”, “Eritrea”, “Djibouti”, “Somalia”, “Kenya”, “Angola”, “Cameroon”, “Central African Republic”, “Chad”, “Democratic Republic of the Congo”, “Equatorial Guinea”, “Gabon”, and “Republic of the Congo” were the main key searching terms. “OR” or “AND” were used separately and in combination as Boolean operators. Notably, to fit the advanced PubMed database, the following search strategy was applied (Appendix 1).

### 2.5 Study selection

Retrieved studies were exported to reference manager software, Endnote version 7, to remove duplicate studies. Four independent reviewers screened the title and abstract. The disagreement was handled based on established article selection criteria. Four independent authors reviewed the abstract and full-text of the articles.

### 2.6 Data extraction

A standardized data extraction format was adopted from the Joanna Briggs Institute (JBI) data extraction format [23] to extract the data. Four authors (YW, AS, EA, and WA) independently extracted all necessary data using this format. The data extraction format included primary author, publication year, country, region, measuring tool, study design, response rate, sample size, and prevalence.

### 2.7 Outcome measurement

The outcome variable of study was perinatal asphyxia, which is defined as the inability of a newborn to initiate and sustain respiration, or a new born with an Apgar score of less than 7 at five minutes after delivery (2). The pooled prevalence was calculated by dividing the total number of perinatal asphyxia in all review studies to the total number of involved fetus and neonates in the study and multiplying by 100 [24].

Perinatal asphyxia = (Number of perinatal asphyxia/number of participants) ∗100.

### 2.8 Quality assessment

The Newcastle-Ottawa Quality Assessment tool [25] was adapted to check the quality of cross-sectional studies in this review [26]. The assessment tool contains 1) representativeness of the sample, 2) sample size, 3) non-respondents and 4) ascertainment of the exposure, 5) independent blind assessment and 6) statistical test) (Appendix 2). Finally, based on this tool, article with a scale of ≥6 out of 10 was considered as good quality.

Each original study was evaluated by four authors independently using this tool. If there were disagreements between those four authors, the consensus was reached by taking the mean score of the four authors. The inter-rater variation of study selection in this study was calculated by using kappa statistics (Appendix 3).

### 2.9 Statistical analysis

Publication bias was checked by funnel plot and more objectively through Begg's and Egger's regression test [27]. Heterogeneity of studies was quantified using the I-Squared Statistic, in which 25, 50, and 75% represented low, moderate and high heterogeneity respectively [28, 29]. Pooled analysis was conducted using a weighted inverse variance random-effects model [30]. Subgroup analysis was done by the study region, and asphyxia measuring tools. Sensitivity analysis was employed to see the effect of single study on the overall estimation. STATA version 14 statistical software was used for meta-analysis.

## 3 Results

### 3.1 Characteristics of reviewed studies

**Figure 1:**
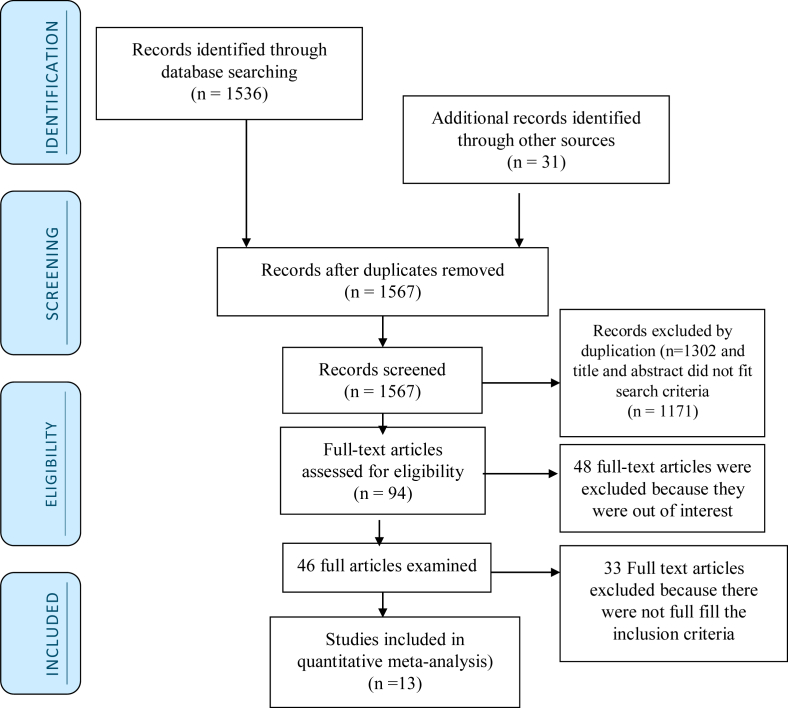
Flowchart to a selection of studies for a systematic review and meta-analysis of the prevalence of perinatal asphyxia in east and central Africa, 2019.

Initially, 1536 records were identified in relation to perinatal asphyxia through PubMed, Google scholar, Africa Index Medicus, Africa Journal Online, EMBASE, and Science Direct databases. Thirty-one records were searched from other sources. From these, 1473 were not considered for further evaluation as a result of duplication and title and abstract did not fit search criteria. From the rest 94 articles, 48 were excluded as a result of not fulfilling our inclusion criteria by reviewing their titles and abstracts. Therefore, 46 full-text articles were accessed and assessed for eligibility based on the inclusion criteria [3, 15, 17, 20, 21, 31, 32, 33, 34, 35, 36, 37, 38, 39, 40, 41, 42, 43, 44, 45, 46, 47, 48, 49, 50, 51, 52, 53, 54, 55, 56, 57, 58, 59, 60, 61, 62, 63, 64, 65, 66, 67, 68, 69, 70, 71]. Finally, 13 studies that fulfilled the eligibility criteria were included in the meta-analysis (Figure 1).

Data for the thirteen eligible studies were extracted and analysed in this study. The pooled prevalence of perinatal asphyxia was estimated by using 76932 newborns. The prevalence of perinatal asphyxia ranges from 3.1% [20] to 32.9% [24]. From thirteen reviewed articles, ten and three articles were form the three East and two Central African countries respectively. Seven of the studies were from Ethiopia [20, 21, 32, 35, 36, 37, 71], two studies were from Kenya [3, 41], one from Tanzania [40], one from Chad [15] and two from Democratic Republic of Congo [43, 44]. Moreover, all thirteen studies were conducted by cross sectional study design (Table 1).

**Table 1:** Descriptive summary of 13 studies included in the meta-analysis of the prevalence perinatal asphyxia in East and Central Africa.

Authors name	Publication Year	Country	Region	Design	Measuring tools	Response rate (%)	Sample size	Prevalence (%)	Quality of study
Sepeku A et al	2011	Tanzania	East Africa	cross-sectional	Apgar score <7	100.0	190	21.1	7
Belachew et al	2017	Ethiopia	East Africa	cross-sectional	Apgar score <7	99.0	368	12.5	6
Ibrahim A et al	2017	Ethiopia	East Africa	cross-sectional	Apgar score <7	100.0	9738	3.1	6
Kibai K et al	2017	Kenya	East Africa	cross-sectional	Apgar score <7	100.0	422	29.1	7
Demisse G et al	2017	Ethiopia	East Africa	cross-sectional	Apgar score <6	100.0	769	12.5	8
Gebreheat G et al	2018	Ethiopia	East Africa	cross-sectional	Apgar score <7	99.7	421	22.1	8
Asfere W et al	2018	Ethiopia	East Africa	cross-sectional	Apgar score <7	100.0	154	29.9	7
Gichogo M et al	2018	Kenya	East Africa	cross-sectional	Apgar score <7	100.0	237	5.1	8
Abkika BM et al	2018	Chad	Central Africa	cross-sectional	Apgar score <7	100.0	7254	5.1	8
Biselele T et al	2013	DRC∗	Central Africa	cross-sectional	Apgar score <6	100.0	902	4.4	7
Mande et al	2018	DRC∗	Central Africa	cross-sectional	Apgar score <7	100.0	612	19.4	8
Alemu A. et al	2019	Ethiopia	East Africa	cross-sectional	Apgar score <7	100.0	262	32.8	7
Abdo RA. et al	2019	Ethiopia	East Africa	cross-sectional	Apgar score <7	100.0	279	15.1	6

DRC∗-Democratic Republic of Congo.

#### 3.1.1 Quality appraisal

The Newcastle-Ottawa Scale quality appraisal criteria established for cross-sectional was used. The studies included in this systematic review and meta-analysis had no low quality. Therefore, all thirteen studies were considered (Table 1).

### 3.2 Meta-analysis

#### 3.2.1 Prevalence of perinatal asphyxia

**Figure 2:**
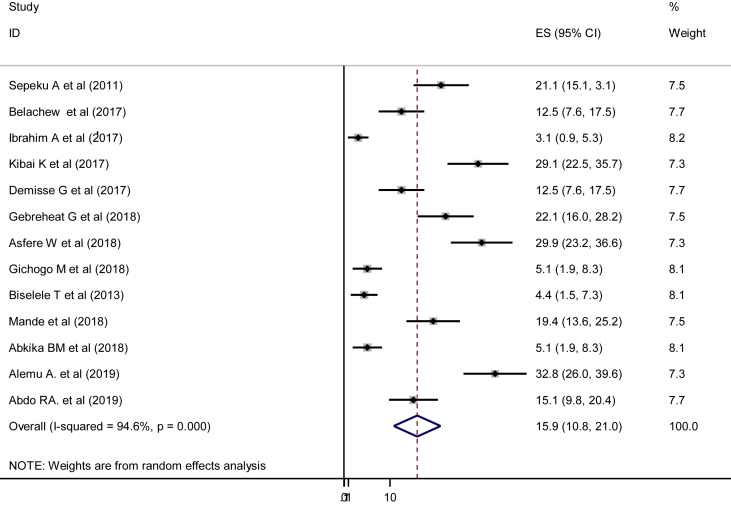
Pooled prevalence of perinatal asphyxia in East and Central Africa, 2019 (n = 13).

**Figure 3:**
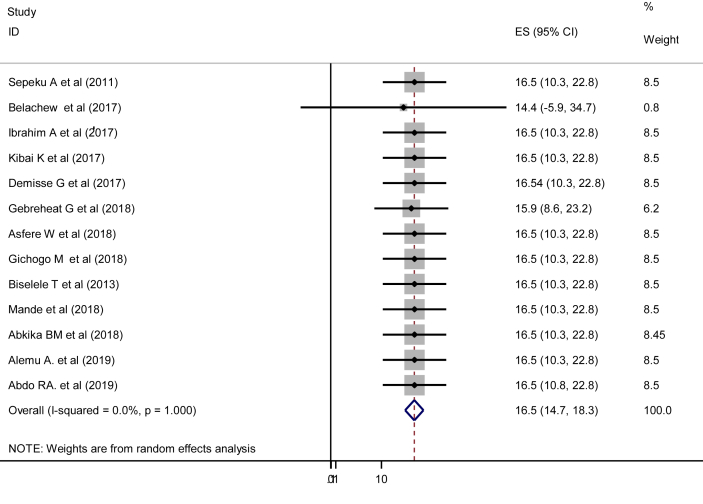
Forest Plot for pooled prevalence of perinatal asphyxia with prediction interval (n = 13).

In the current study, the pooled prevalence of perinatal asphyxia was 15.9% (95% CI: 10.8, 21.0%). Severe heterogeneity was observed across the studies (I^2^ = 94.6, p = 0.000) (Figure 2). The pooled prevalence of perinatal asphyxia with a prediction interval in this study was 16.5% (95% CI:14.7, 18.3%) (Figure 3).

#### 3.2.2 Subgroup analysis

As a result of substantial heterogeneity, we performed subgroup analysis based on the region and measuring tools. In this regard, the prevalence was higher in East Africa, 18.0 % (95%CI:11.4, 26.7% [I^2^ = 96.0, p = 0.000]) as compared with Central Africa, 9.1 % (95%CI:2.0, 16.2% [I^2^ = 90.8, P = 0.000]). The prevalence of asphyxia, which was determined via Apgar scale of <7 was higher than the prevalence which was measured by Apgar scale of <6 (Table 2).

**Table 2:** Subgroup analysis of prevalence of perinatal asphyxia in East and Central Africa.

Variables	Characterstics	Estimates (95%CI)	I^2^ tests with p-value
Region	East Africa	18.0 % (11.4, 26.7%)	I^2^ = 96.0, p = 0.000
	Central Africa	9.14 % (2.0, 16.2%)	I^2^ = 90.8, P = 0.000
Measuring scale	Apgar score <7	17.4 % (11.3, 23.5%)	I^2^ = 96.0, P = 0.000
	Apgar score <6	8.2 % (0.3, 16.1%)	I^2^ = 86.9, p = 0.006

A random-effect model was employed to estimate the pooled prevalence of perinatal asphyxia. Different factors associated with the heterogeneity such as publication date, sample size and response rate were investigated using multivariate meta-regression models. From these variables, none of them were statistically significant (Table 3).

**Table 3:** Related factors with heterogeneity of perinatal asphyxia prevalence in the current meta-analysis.

Variables	Coefficient	P-value
Publication years	2.9 (0.6, 4.8)	0.8
Sample size	-0.1 (-1.5, 0.6)	0.4
Response rate	-9.5 (-14.5, -0.7)	0.4

##### 3.2.2.1 Publication bias

**Figure 4:**
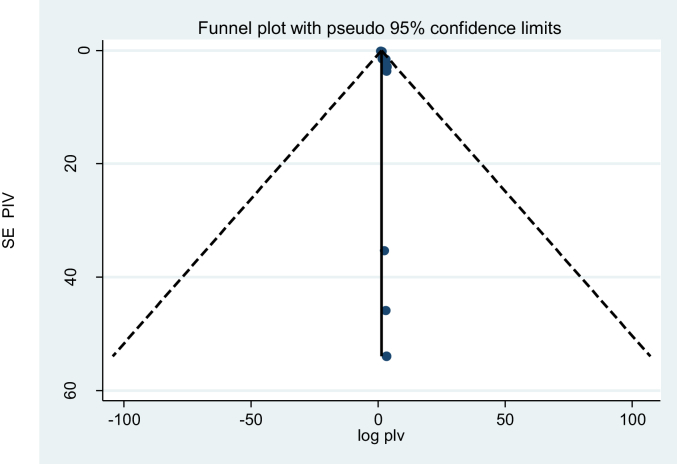
Funnel plot for publication bias, logprop or lnp (log of proportion) represented in the X-Axis and standard error of log proportion in the Y-Axis.

**Figure 5:**
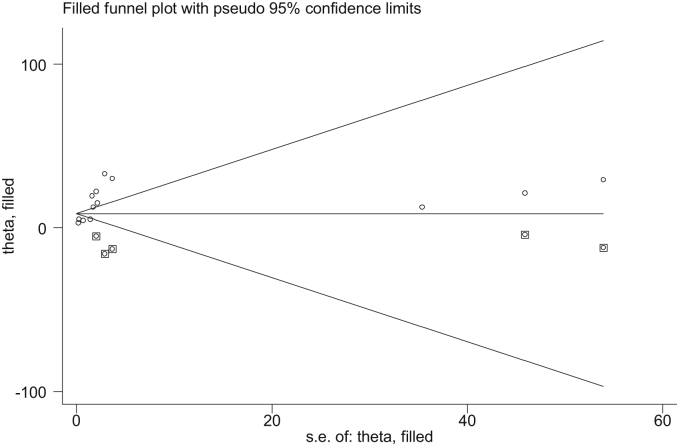
The trim fill analysis showed the pooled prevalence when the unpublished studies are filled.

A funnel plot showed asymmetrical distribution (Figure 4). The result of the Egger test was also statistically significant with Bo = 1.9 and p = 0.000. To see publication bias further, trim fill analysis was done, and five studies were filled. In this analysis, the pooled prevalence of perinatal asphyxia was 9.7% (95% CI: 6.2, 12.3%). But the confidence interval indicated that there is no difference in the pooled prevalence of perinatal asphyxia (Figure 5).

##### 3.2.2.2 Sensitivity analysis

**Figure 6:**
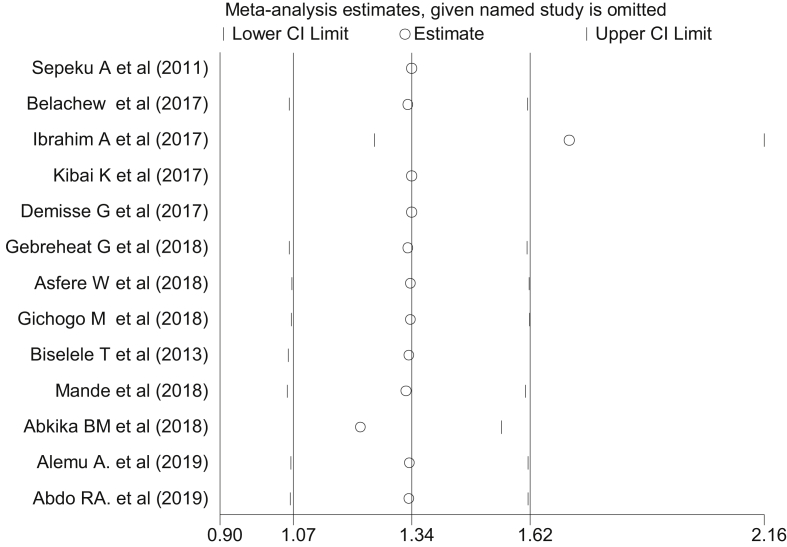
The sensitivity analysis showed the pooled prevalence when the studies omitted step by step.

Among all thirteen reviewed studies in the current analysis, the study conducted by Ibrahim et al [20] had shown an impact on the overall estimation (Figure 6). The pooled prevalence of perinatal asphyxia in terms of step by step omission of article is indicated in (Table 4).

**Table 4:** The sensitivity analysis to estimate of the prevalence of perinatal asphyxia in East and Central African countries.

Study omitted	Prevalence (95%CI)
Sepeku A et al, 2011	15.5 (10.3, 20.7)
Belachew et al, 2017	16.2 (10.8, 21.7)
Ibrahim A et al, 2017	17.1 (11.6, 22.5)
Kibai K et al, 2017	14.8 (9.8, 19.8)
Demisse G et al, 2017	16.2 (10.8, 21.7)
Gebreheat G et al, 2018	15.4 (10.2, 20.6)
Asfere W et al, 2018	14.8 (9.8, 19.7)
Gichogo M et al, 2018	16.9 (11.3, 22.5)
Biselele T et al, 2013	16.9 (11.4, 22.6)
Mande et al, 2018	15.6 (10.6, 20.9)
Abkika BM et al, 2018	16.9 (11.3, 22.5)
Alemu A. et al, 2019	14.5 (9.7, 19.3)
Abdo RA. et al, 2019	16.0 (10.6, 21.4)

## 4 Discussion

This study revealed that the pooled prevalence of perinatal asphyxia among newborns was 15.9%. This finding was lower than the study conducted in Western Africa (30.0%) [72]. Lower level of asphyxia in the present study as compared with the study conducted in West Africa might be due to variation in study population. In the present study, the study participants were all newborns, but the study participants in West Africa were all admitted neonates. West African countries are better equipped with modern health facilities as compared East and Central Africa countries health facilities. On the contrary of this evidence, the prevalence of perinatal asphyxia is higher in West Africa as compared with East and Central Africa countries [18]. Therefore, further investigation should be done to identify the main reasons for this variation across the regions.

On the other corner, the current pooled prevalence of perinatal asphyxia is higher than the study conducted in South Africa (2.6%) [9]. The lower level of perinatal asphyxia in Southern Africa as compared with the current study is due to the development and implementation of the South African Neonatal Resuscitation programme [73]. This program in South Africa have brought a strong association between the establishment of an effective audit process, and improvement of the quality of maternal health services and perinatal mortality rates [66, 74]. Additional reasons for high level of perinatal asphyxia in the present study as compared with Southern Africa is the presence of low levels of reproductive health services utilization (facility deliveries, skilled delivery assistance and 4^+^ antenatal visits) in East and Central Africa as compared to South africa [18].

Subgroup analysis pointed out that the prevalence of perinatal asphyxia was 18.0 % in East Africa region and the prevalence of birth asphyxia was 9.1% in Central Africa region. This indicated that substantial heterogenecity of the prevalence of perinatal asphyxia in East and Central Africa countries. Such difference might be due to obstetrics service delivery (coverage, equity and quality) variation across East and Central Africa countries. As the evidence pointed out that obstetrics coverage, equity and quality gaps brings adverse maternal and neonatal outcomes [75, 76]. The other reason for the lower prevalence in Central Africa might be due to only three studies were included in the review process.

As a result of high burden of asphyxia, the researchers should pool the risk factors of perinatal asyphaxia in order to design the preventive mechanism of modifiable factors of perinatal asphyxia. In general, the data in this report will provide useful information for health planners and politicians involved in health care provision.

This study might be subjected to different limitations. The first limitation of the study was only English articles or reports were considered to carry out the analysis. Even though the quality of each study was assessed by using The Newcastle-Ottawa Scale, inter author bias might be occurred on leveling of the scale of each article. Reviewing of different characterstics of involved cases with different sampling methods was also the other limitation of this study. The last but not the least limitation of the current study is availability of limited numbers of the studies.

## 5 Conclusion

There was a high rate of perinatal asphyxia. It had also sources of heterogeneity in terms of geographical setting and measuring tools. Therefore, there is an alarm for responsible bodies to decrease the high level of this problem in the region.

## Declarations

### Author contribution statement

All authors listed have significantly contributed to the development and the writing of this article.

### Funding statement

This research did not receive any specific grant from funding agencies in the public, commercial, or not-for-profit sectors.

### Competing interest statement

The authors declare no conflict of interest.

### Additional information

No additional information is available for this paper.

## Figures and Tables

**Table:** 

Search #	Query	Items found
#1	perinatal	119521
#2	(birth) OR parturition	527962
#3	(newborn) OR neonate	249893
#4	(asphyxia) OR (suffocation) OR respiratory distress syndrome	88236
#5	Search (Tanzania AND Burundi AND Rwanda AND Uganda AND Sudan AND Ethiopia AND Eritrea AND Djibouti AND Somalia AND Kenya AND Angola AND Cameroon AND Central African Republic AND Chad AND Democratic Republic of the Congo AND Equatorial Guinea AND Gabon AND Republic of the Congo)	279
#6	#1 AND #2 AND #3 AND #4 AND #5	11

**Table:** 

		Selection				Comparability		Outcome		Total score
Study	Quality assessor	Representativeness of sample (∗, ∗)	Sample size (∗)	Non-respondents (∗)	Ascertainment (∗∗)	Study controls for most important factor (∗)	The study control for any additional factor (∗)	Assessment of the outcome (∗)	Statistical test (∗)	
Sepeku A et al.	YW	1	1	1	1	1	1	1	1	7
	AY	1	1	1	1	1	1	1	1	
	EA	1	0	0	1	1	1	1	0	
	WA	1	1	1	1	1	0	1	1	
Belachew et al.	YW	1	0	0	2	1	1	1	0	6
	AY	0	1	1	1	1	1	1	1	
	EA	1	0	1	1	1	1	1	0	
	WA	0	1	1	0	1	1	1	0	
Ibrahim A et al.	YW	1	1	1	0	1	0	0	1	6
	AY	0	1	1	1	1	0	0	1	
	EA	1	1	1	1	1	1	1	0	
	WA	1	0	1	2	1	0	0	1	
Kibai K et al.	YW	1	1	1	1	1	1	1	1	7
	AY	1	1	1	1	1	1	1	0	
	EA	1	0	0	1	1	1	1	1	
	WA	1	1	1	1	1	0	1	1	
Demisse G et al.	YW	1	1	1	1	1	1	1	1	8
	AY	1	1	1	1	1	1	1	1	
	EA	2	1	0	1	1	1	0	1	
	WA	2	1	1	1	1	1	1	1	
Gebreheat G et al.	YW	2	0	1	2	1	1	1	0	8
	AY	2	1	1	2	1	0	1	1	
	EA	2	1	1	1	1	1	1	0	
	WA	1	1	1	0	1	1	1	1	
Asfere W et al.	YW	1	1	1	1	1	1	1	1	7
	AY	1	1	1	1	1	1	1	1	
	EA	1	0	0	1	1	1	1	0	
	WA	1	1	1	1	1	0	1	1	
Gichogo M et al.	YW	2	0	0	2	1	1	1	0	8
	AY	2	1	1	2	1	0	1	1	
	EA	2	1	1	1	1	1	1	0	
	WA	1	1	1	2	1	1	1	1	
Abkika BM et al.	YW	1	1	1	1	1	0	1	1	8
	AY	1	1	1	1	1	1	1	1	
	EA	1	1	1	2	1	1	1	1	
	WA	1	1	1	2	1	1	0	1	
Biselele T et al.	YW	1	1	1	1	1	1	1	1	7
	AY	1	1	1	1	1	1	1	1	
	EA	1	1	1	1	1	1	1	0	
	WA	1	1	1	1	1	0	1	1	
Mande et al.	YW	1	1	1	1	1	0	1	1	8
	AY	2	1	1	1	1	1	1	0	
	EA	2	1	1	2	1	1	0	1	
	WA	1	1	1	2	1	1	0	1	
Alemu A. et al.	YW	1	1	1	1	1	1	1	1	7
	AY	2	1	1	2	1	1	1	0	
	EA	1	1	0	1	1	1	0	1	
	WA	2	1	1	1	1	1	1	1	
Abdo RA. et al.	YW	1	0	1	0	1	1	1	1	6
	AY	1	1	1	1	1	0	1	0	
	EA	1	0	1	1	0	1	1	1	
	WA	0	1	1	1	1	1	1	0	

**Table:** 

Item	Kappa	SEM	95% CI
Representativeness of sample	0.37	0.07	(1.00, 1.30)
Sample size	0.03	0.06	(0.69, 0.91)
Non-respondents	0.16	0.05	(0.77, 0.96)
Ascertainment	0.17	0.07	(1.00, 1.30)
Study controls for most important factor	0.02	0.02	(0.94, 1.02)
The study control for any additional factor	0.01	0.06	(0.65, 0.89)
Assessment of the outcome	0.02	0.05	(0.74, 0.95)
Statistical test	0.16	0.06	(0.56, 0.82)
Overall	0.30	0.22	(0.17, 0.63)
